# Alcohol and substance use and associated risk factors in nursing undergraduates at a South African university

**DOI:** 10.4102/hsag.v30i0.2973

**Published:** 2025-11-20

**Authors:** Gaotswake P. Kovane, Pat M. Mayers

**Affiliations:** 1 School of Nursing, Faculty of Community and Health Sciences, University of the Western Cape, Cape Town, South Africa; 2 School of Nursing, North-West University, NuMIQ Focus Area, Mafikeng, South Africa

**Keywords:** alcohol, nursing students, risk factors, substance use, South Africa

## Abstract

**Background:**

The use of substances by university nursing students is a significant public health challenge and may impact their professional conduct and compromise the quality of patient care.

**Aim:**

The study aimed to investigate alcohol and substance use by undergraduate nursing students, and the associated risk factors, at a university in the Western Cape province, South Africa.

**Setting:**

The study was conducted at a university in the Western Cape province, South Africa.

**Methods:**

A quantitative, descriptive survey design was used. A convenience sample of second-, third- and fourth-year nursing students completed a self-report online questionnaire. Descriptive statistics, Chi-square tests and multivariate linear regression were used to analyse the data.

**Results:**

A total of 212 questionnaires were completed. Most respondents (81%; *n* = 171) were female. The substances most used were tobacco (24.1%), alcohol (64.6%) and cannabis (marijuana) (23.7%). Few students had used ‘hard’ recreational drugs. Female students were more likely to have used alcohol and drugs over the 12 months preceding the study.

**Conclusion:**

To limit alcohol and drug use of nursing students, nursing education institutions need to increase efforts to raise awareness, include relevant curriculum content and provide appropriate support.

**Contribution:**

This study highlights the substance use risks and behaviours of undergraduate nursing students. Nursing students, as future health professionals, need to be empowered to make informed choices about the use of alcohol and other substances and need to be supported by university policies, appropriate education and counselling services.

## Introduction

Substance use is a worldwide public health problem. Globally, in 2021, one in every 17 people aged 15–64 had used a drug in the previous 12 months, a 23% increase (United Nations Office on Drugs and Crime 2023). In 2019, alcohol caused 2.6 million deaths, and psychoactive drugs accounted for nearly 600 000 deaths worldwide (World Health Organization 2024). Substance use and abuse increase a country’s economic burden (Manthey et al. 2021) and are associated with automobile accidents and critical injuries, resulting in increased medical costs, morbidity and mortality (Kameg et al. 2020; Ncube, Cheteni & Sindiyandiya 2016). Substance use has a significant impact on mental health, often co-occurring with mental health conditions and worsening their health outcomes (World Health Organization 2024). Illicit substance use has increased substantially in South Africa (Mutai et al. 2024; Peltzer & Phaswana-Mafuya 2018). In the Western Cape province, high substance use and abuse among youth have been reported, with past 3-month drug use of 7.1% (Peltzer & Phaswana-Mafuya 2018).

Student success in higher education is dependent not only on their academic ability but also on their physical and mental wellness. Higher education institutions worldwide are recording increasing levels of student anxiety and related mental disorders and substance use (eds. Scherer & Leshner 2021). Student nurses have tended to abuse drugs without a prescription, which has been associated with binge drinking, with detrimental effects of weight gain (Sousa et al. 2020). Reported reasons for substance use among nursing students include stress management (Ruth-Sahd & Schneider 2022), peer influence (Gupta, Gupta & Rozatkar 2021), living in hostel accommodation and away from home (Van Zyl et al. 2015), challenges of their clinical placements (Chaabane et al. 2021; Ching et al. 2020) and a desire to experiment (Silva et al. 2014). Protective factors for substance use and misuse include active religious observance (Zanetti, Cumsille & Mann 2019) and family values and support (Soliman et al. 2022). In South African universities, the main substances used by students are alcohol, cannabis and ecstasy (Blows & Isaacs 2022; Chen et al. 2023).

The use of substances by healthcare professionals may impact their professional conduct and compromise the quality of healthcare services provided, posing a risk to the public (Panthee et al. 2017). Studies with health sciences students have reported substance use in countries that include, among others, the US (United States) (Ruth-Sahd & Schneider 2022), Brazil (Dos Santos et al. 2019), Cameroon (Mbanga et al. 2018), Ethiopia (Alebachew et al. 2019) and Spain (Rabanales Sotos et al. 2015). Although the substance use prevalence rate for health sciences students is generally lower than for other students, it remains of concern, as it has a negative impact on student performance (Meda et al. 2017) and general health (Mbanga et al. 2018) and may lead to risky conduct in future healthcare workers (Van Zyl et al. 2019).

The substances commonly used by nursing students are alcohol, glue and other solvents, waterpipe, cocaine, tobacco, cannabis, cigarettes, sedatives and tranquilisers without prescription (Colomer-Pérez et al. 2019; Sousa et al. 2020; Van Zyl et al. 2019). The use of alcohol and drugs may not only impede academic performance but also affect patient care (Van Zyl et al. 2019). Chronic drug and alcohol abuse can compromise patient care, as individuals struggling with substance use may be unable to make sound clinical decisions (Tejedor-Cabrera & Cauli 2019). This is a concern for the healthcare workforce and health sciences education.

### Aim of the study

The study aimed to investigate alcohol and substance use by undergraduate nursing students, and the associated risk factors, at a university in the Western Cape province, South Africa.

## Research methods and design

A quantitative, descriptive survey design was used. All second-, third- and fourth-year nursing students, aged 18 or older, registered for the 2022 academic year in the Bachelor of Nursing programme at the selected university were eligible for inclusion (total population 541). All students who were willing to participate in the study formed a convenience sample. A self-report structured questionnaire, adapted from the Core Alcohol and Drug Survey (Presley, Meilman & Lyerla 1994), was used. This tool investigates the type, extent, risks and outcomes of alcohol and other drug use among students in higher education institutions. The questionnaire was adapted with minimal changes made only to terminology more familiar to South African students. Content validity was ensured by aligning the questions with the study objectives. The questionnaire was pretested with 10 students who were not included in the main study. The questionnaire required no more than 30 min to complete.

### Data collection and analysis

Data were collected over 3 days in September 2022 through an anonymised web-based questionnaire, using the Research Electronic Data Capture (RedCap) system. Arrangements were made with lecturers to present the study to the students after lecture sessions. Students who met the inclusion criteria and were interested in participating were informed by the first author. After a student had given written informed consent, they were sent a link to the anonymous online survey.

Data were analysed using the Statistical Package for Social Sciences (SPSS) v.28 statistical package for Windows. The descriptive data are reported using numbers, percentages and frequency distributions (5% significance and 95% confidence interval). Chi-square tests were performed to determine associations between substance use by nursing students and variables such as age and religious affiliation. Multivariate linear regression analysis was performed to determine relationships between variables.

### Ethical considerations

Ethical approval for the study was obtained on 02 June 2022 from the University of the Western Cape Research Ethics Committee (HSSREC # HS21/10/82). The university registrar and the head of the nursing school granted permission to access the nursing students. By ensuring that participant privacy and confidentiality were upheld and safeguarded throughout the study, the researcher ensured that ethical standards were maintained. Informed consent was obtained from all students who met the inclusion criteria and voluntarily agreed to participate. No identifying information was obtained; anonymised study numbers were assigned, ensuring that no research data could be linked to respondents. All records were password-protected and electronically stored. Only the researchers and the statistician had access to the study data.

## Results

A total of 212 questionnaires were received. Most respondents were female (81%, *n* = 171), and one did not indicate gender. A total of 94% of the students were single, and 81% identified with the Christian religion (Table 1).

**TABLE 1:** Socio-demographic characteristics of respondents.

Variables	Second-year		Third-year		Fourth-year		Total	
	*n*	%	*n*	%	*n*	%	*n*	%
**Gender**								
Male	8	17.8	19	25.3	13	14.4	40	18.9
Female	38	84.4	57	76.0	76	84.4	171	80.7
Not stated	-	-	-	-	-	-	1	-
Total	46	-	76	-	89	-	212	-
**Religion**								
Christianity	39	86.7	60	80.0	71	78.9	170	80.2
Islam	1	2.2	9	12.0	15	16.7	25	11.8
African traditional	2	4.4	4	5.3	1	1.11	7	3.3
Other	3	6.7	2	2.7	4	4.4	9	4.3
Not stated	-	-	-	-	-	-	1	-
Total	-	-	-	-	-	-	212	-
**Marital status**								
Single	44	97.8	70	93.3	85	94.4	199	93.9
Married	0	-	4	5.3	5	5.6	9	4.3
Separated	0	-	1	1.3	0	-	1	0.5
Divorced	0	-	0	-	2	2.2	2	0.9
Not stated	-	-	-	-	-	-	1	-
**Total**	-	-	-	-	-	-	212	-

*Source*: Adapted from Kovane, G.P., 2023, ‘An investigation of alcohol and drug use and possible risk factors amongst nursing students at a university in the Western Cape’, Master’s thesis, University of the Western Cape, Cape Town, viewed 08 August 2024, from https://uwcscholar.uwc.ac.za/items/c3cf1d70-c976-4c45-8756-d968c4cf1c79

Boredom (50%), ‘the influence of friends’ (42%) and ‘to forget problems’ (41%) were the predominant reasons for students’ use of substances. Other reasons included ‘to calm down when angry’ (33%), ‘to sleep comfortably’ (38%), ‘to experiment for fun’ (39.8%) and family influence (26%) (Table 2).

**TABLE 2:** Reasons for substance use.

Reasons for substance use	Percentage					
	Strongly agree	Agree	Neutral	Disagree	Strongly disagree	Don’t know
Trying out and/or for fun	19.90	19.90	17.48	7.77	18.93	16.02
Boredom	19.42	30.58	13.11	5.34	16.99	14.56
Friends’ influence	9.71	15.05	17.48	15.53	26.70	15.53
To forget problems	11.96	14.35	14.35	15.31	28.71	15.31
No specific reason	11.71	15.61	22.93	8.29	22.93	18.54
To calm down when angry	8.17	10.10	14.90	17.79	30.29	18.75
To sleep comfortably	11.54	11.06	14.90	16.83	28.37	17.31
Family influence	7.25	6.28	12.08	17.87	39.13	17.39

*Source*: Adapted from Kovane, G.P., 2023, ‘An investigation of alcohol and drug use and possible risk factors amongst nursing students at a university in the Western Cape’, Master’s thesis, University of the Western Cape, Cape Town, viewed 08 August 2024, from https://uwcscholar.uwc.ac.za/items/c3cf1d70-c976-4c45-8756-d968c4cf1c79

Alcohol and drug use in the past 12 months.

Respondents were asked to reflect on their alcohol and substance use in the 12 months preceding the survey (Table 3).

**TABLE 3:** Substance use in the 12 months preceding the survey.

Frequency of use	Tobacco		Alcohol		Marijuana		Cocaine	
	*n*	%	*N*	%	*n*	%	*n*	%
**Second year**								
Did not use	29	64.4	13	28.9	36	80.0	44	97.8
Rarely	6	13.3	8	17.8	4	8.9	1	2.2
Sometimes	3	6.7	18	40.0	1	2.2	0	0.0
Often	6	13.3	6	13.3	3	6.7	0	0.0
Always	1	2.2	0	0.0	1	2.2	0	0.0
Total	45	-	45	-	45	-	45	-
**Third year**								
Did not use	51	67.0	24	32.0	51	67.1	74	98.7
Rarely	6	8.0	16	21.3	10	13.3	1	1.3
Sometimes	4	5.3	17	22.4	7	9.3	0	0.0
Often	9	12.0	18	24.0	6	8.0	1	1.3
Always	6	8.0	1	1.3	2	2.7	0	-
Total	76	-	76	-	76	-	76	-
**Fourth year**								
Did not use	78	85.7	38	41.8	75	82.4	88	96.7
Rarely	6	6.6	12	13.2	7	7.7	3	3.3
Sometimes	1	1.1	27	29.7	5	5.5	0	0.0
Often	1	1.1	14	15.4	2	2.2	0	0.0
Always	5	5.5	0	0.0	2	2.2	0	0.0
Total	91	-	91	-	91	-	91	-

*Source*: Adapted from Kovane, G.P., 2023, ‘An investigation of alcohol and drug use and possible risk factors amongst nursing students at a university in the Western Cape’, Master’s thesis, University of the Western Cape, Cape Town, viewed 08 August 2024, from https://uwcscholar.uwc.ac.za/items/c3cf1d70-c976-4c45-8756-d968c4cf1c79

The majority of students (74.5%) reported not using tobacco products in the 12 months preceding the survey. Students in their second year were least likely to smoke (2.2%), and most (*n* = 163; 77%) had not used cannabis. Alcohol was more commonly used; 64.6% had used alcohol in the year preceding the survey. Very few respondents had used psychedelic drugs; 97.6% had never used amphetamines (diet pills, speed), sedatives, hallucinogens (LSD, PCP) or opiates (e.g. heroin, smack). Most respondents (*n* = 206; 97.6%), proportionately evenly distributed in all year levels, had never used cocaine. There were no reports of the usage of steroids, designer drugs or inhalants such as glue, solvents or gas.

Table 4 lists possible risk factors that affect substance use among the university nursing students during the 12-month and 30-day periods preceding the study.

**TABLE 4:** Multivariate regression analysis on the consumption of alcohol and drugs in the past year, past 30 days and general use.

Variable	Q17_Past year†			Q18_Past 30 days†			Q19_How often‡		
	Coef.	s.e.	*P* > *t*	Coef.	s.e.	*P* > *t*	Coef.	s.e.	*P* > *t*
**Classification (fourth year)**									
Second year	0.23	0.14	0.11	0.37	0.14	0.01	0.21	0.14	0.13
Third year	0.11	0.10	0.27	0.02	0.10	0.87	0.07	0.10	0.49
**Age (years)**									
20–21	0.14	0.15	0.33	0.21	0.15	0.16	0.12	0.15	0.40
25+	0.28	0.19	0.15	0.55	0.19	0.00	0.26	0.19	0.17
**Religion (Traditional)**									
Christianity	0.01	0.25	0.97	0.31	0.24	0.21	0.04	0.24	0.88
Islam	0.04	0.27	0.89	0.07	0.27	0.79	0.10	0.27	0.71
Other	0.18	0.34	0.60	0.15	0.33	0.65	0.03	0.33	0.94
**Gender (Female)**									
Male	−0.07	0.11	0.54	0.06	0.11	0.59	−1.15	0.11	-
**Res (off campus)**									
On-campus	0.07	0.10	0.49	0.06	0.10	0.54	0.11	0.09	0.24
**Working (Partial)**									
Yes, full time	0.05	0.22	0.83	0.08	0.22	0.71	0.28	0.22	0.20
Yes, part-time	0.02	0.17	0.89	0.35	0.16	0.04	0.12	0.17	0.52
**Alcohol Possession (available)**									
Not have available	−0.01	0.12	0.90	0.10	0.11	0.38	0.05	0.113	0.64
**Status**									
Part-time (1–11 credits)	−0.12	0.32	0.70	−0.45	0.31	0.15	0.63	0.31	0.04
**Prevention (Yes)**									
No	−0.09	0.15	0.57	−0.30	0.15	0.05	0.29	0.15	0.05
**Change in alcohol use**									
Decreased	0.05	0.13	0.72	−0.12	0.12	0.34	0.001	0.12	0.99
I have not used alcohol	0.06	0.15	0.70	−0.14	0.14	0.33	0.07	0.14	0.65
Increased	−0.06	0.15	0.67	−0.07	0.15	0.64	0.11	0.15	0.45
**Change in drugs (Remained unchanged)**									
Decreased	0.13	0.19	0.48	−0.08	0.19	0.65	0.22	0.19	0.24
I have not used drugs	0.06	0.16	0.70	−0.14	0.16	0.37	0.42	0.16	0.01
Increased	0.13	0.23	0.58	−0.38	0.23	0.10	0.19	0.23	0.40
_Cons	0.72	0.63	0.26	1.72	0.62	0.006	1.94	0.61	0.002

*Source*: Adapted from Kovane, G.P., 2023, ‘An investigation of alcohol and drug use and possible risk factors amongst nursing students at a university in the Western Cape’, Master’s thesis, University of the Western Cape, Cape Town, viewed 08 August 2024, from https://uwcscholar.uwc.ac.za/items/c3cf1d70-c976-4c45-8756-d968c4cf1c79

Note:

† , 1 = More often, 0 = Never consumed or less often;

‡ , generally.

s.e., standard error; Coef., coefficient; _Cons, constant coefficient.

Second-year students were 23% more likely to have used alcohol in the 12 months preceding the survey (*B* = 0.23, *p* < 0.05). This group was 21% more likely to have used drugs and alcohol than other year groups (*B* = 0.21, *p* > 0.05), however, in the 30 days preceding the survey, these students were 37% less likely to have used substances (*B* = −0.37, *p* > 0.05) in comparison to the other senior-level students (*B* = 0.02, *p* > 0.05).

Students older than 25 years were 28% more likely to have used alcohol in the 12 months preceding the survey (*B* = 0.28, *p* < 0.05), compared with students in the 20–21-year age group (*B* = 0.14, *p* > 0.05). In the 30 days preceding the survey, the older students were twice as likely to use substances (*B* = 0.55, *p* > 0.05) than younger students (*B* = 0.21, *p* > 0.05).

Regarding religion, Muslim (*B* = −0.04, *p* > 0.05) or Christian students (*B* = −0.01, *p* > 0.05) were less likely to use alcohol in the 12 months preceding the survey. In the 30 days preceding the survey, compared to Muslim and Christian students, those with other or no religious affiliations were 15% more likely to have used drugs and alcohol (*B* = 0.15, *p* > 0.05).

*Gender:* Male respondents were 7% less likely than females to have used alcohol and drugs in the 12 months preceding the survey (*B* = −0.07, *p* > 0.05). Although male respondents were generally 15% less likely to use alcohol and drugs, they were more likely to have used alcohol and drugs (*B* = 0.06, *p* > 0.05) in the 30 days preceding the survey. This may have been because of factors such as social or sporting events, aspects which were not included in the questionnaire.

Students were less likely to use drugs and alcohol if they stated that they would rather not drink at parties or other events (*B* = −0.01, *p* > 0.05).

## Discussion

In this study, nursing students reported that they used substances to manage their problems, which has also been reported in other studies. In Cameroon, nursing students used alcohol and other substances to cope with the pressures of a heavy study workload and their clinical care responsibilities (Mbanga et al. 2018). Stress alleviation as a reason for using substances has been reported in Ethiopia (Patel et al. 2016) and Malawi (Baluwa et al. 2021). The influence and pressure of students’ social groups to use substances, found in our study, was also reported by Fernández-García et al. (2020). Du Preez, Pentz and Lategan (2016), however, found that conforming to a peer group was not a predictor of alcohol consumption in university students. The context and culture of the peer group may be stronger than the individual’s personal preferences, thus acting as a strong influence on behaviour.

Male and female students differed in their use of substances. In the South African general population, women are far less likely to use alcohol than men (Peltzer & Phaswana-Mafuya 2018); however, in the student population, males are more likely to use substances than females (Shuro & Waggie 2024). Our study, on a single university nursing student group, found, however, that males were less likely than females to use alcohol and drugs over the 12 months preceding the study. Similar findings in studies with nursing students were reported in Brazil (Monteiro et al. 2018) and in Nigeria (Charles et al. 2021).

The high use of cannabis in this study has been reported in studies in other South African universities (Jain et al. 2018; Ramdhani 2017) and worldwide, including Nepal (Panthee et al. 2017), Cameroon (Mbanga et al. 2018) and Europe (Colomer-Pérez et al. 2019). The recent legislation, which decriminalises and regulates the use of cannabis for private purposes, may change the future pattern of use (South Africa 2024).

Family dynamics, relations and management have an important influence on adolescents’ use of substances (Muchiri & Dos Santos 2018). The family can be an essential resource in sensitising young people to the harmful effects of substance use and abuse (Ajayi & Somefun 2020). In this study, more than half (57%) of the respondents indicated that there was no negative family influence on their use of alcohol and drugs. Medical students in North Karnataka, India whose parents had a history of tobacco use were likely to use tobacco (Patel et al. 2016). A Nigerian study found that students, whose parents were alcohol drinkers, were also at risk of using alcohol (Charles et al. 2021).

In this study, two main reasons for the use of substances were experimentation for fun (39.8%) and to alleviate boredom (50%). Boredom has been reported as playing an important role in contributing to substance use (Magidson et al. 2020; Weybright et al. 2015). Recreational drug use, reported in this study, has been recognised as an increasingly concerning public health issue, particularly in low-middle-income countries (Ajayi & Somefun 2020; Weybright et al. 2016).

Several studies have documented the favourable impact of spiritual beliefs, religious practices and religion on substance use (Cole et al. 2020; Palamar, Kiang & Halkitis 2014; Zanetti et al. 2019). In our study, Christian and Muslim students were less likely to use alcohol and drugs in the 12 months before the study commenced, compared to students from other religions or non-religious students.

### Implications and recommendations

Although universities have policies in place regarding substance use, the findings of this study indicate that more investment is needed in mental health and wellness programmes and supportive measures to curb the rate of alcohol and drug use among university students. Measures to curb substance use by nursing students should include raising awareness among students and parents (Abazid et al. 2023), counselling (Moagi & Van der Wath 2021), the provision of accessible, confidential counselling services in person and online, walk-in crisis support services and support from suitably trained nurse mentors. Access to alcohol at university events should be regulated (Temper 2021). Nursing curricula should include substance use-related content (Finnell et al. 2018), which applies not only to patient care but also to students’ lifestyles. Nursing education institutions should establish a strong support system to equip students with effective coping skills (Chaabane et al. 2021). Nursing students, who often have full and stressful programmes (Boulton & O’Connell 2017), may need encouragement to have a healthy work-life balance and participate in the many extra-curricular activities on a university campus.

### Limitations

This study has limited generalisability as it was conducted with a convenience sample of nursing students from one university, and as a descriptive study, it describes only the current state of the phenomenon. A self-reported survey depends on the honesty of the respondents, as they may provide socially desirable answers or intentionally mislead. Surveys limit the breadth and depth of the data and may miss the nuanced insights of qualitative approaches. Several other ‘street drugs’, or combinations of drugs, widely available in the Western Cape, were not included in the questionnaire (Western Cape Government 2019).

## Conclusion

Nursing students in this study used mainly tobacco, alcohol and cannabis, with very few having experimented with or used the hard recreational or psychedelic substances. Further research is required not only to determine the prevalence of substance use but also how it affects nursing practice and education, as well as how to assist and educate nursing students who use substances. The use of substances may interfere with cognitive function, resulting in students’ poor academic performance, absenteeism and difficulties at clinical placements. Substance use may result in poor judgements being made in critical situations, leading to management and/or medication errors, which negatively affect quality patient care. Students should be empowered to make informed choices about the use of alcohol and other substances. Universities need to have clear policies and strategies to educate students on substance use and provide support for future health professionals. These may include awareness-raising strategies, inclusion of relevant content in curricula, clear guidelines regarding the use of substances on campus and relevant empathic mental health support structures.
